# Small RNA existed in commercial reverse transcriptase: primary evidence of functional small RNAs

**DOI:** 10.1007/s13238-014-0116-2

**Published:** 2014-11-22

**Authors:** Jie Xu, Xi Chen, Donghai Li, Qun Chen, Zhen Zhou, Dongxia Hou, Jin Wang, Qipeng Zhang, Ke Zen, Chen-Yu Zhang

**Affiliations:** Jiangsu Engineering Research Center for microRNA Biology and Biotechnology, State Key Laboratory of Pharmaceutical Biotechnology, School of Life Sciences, Nanjing University, Nanjing, 210093 China

**Table:** 

SMALL RNA IN REVERSE TRANSCRIPTASE: TOO SMALL TO BE FOUND	
Significant background in no-template or no-primer RT-PCR is frequently noted. However, the underlying mechanism is still unclear. In this issue, Jie Xu and colleagues reveal that the commercial reverse transcriptases contain small RNAs. In AMV reverse transcriptase, they detected microRNAs derived from the virus host chick or other birds. This surprising finding at least partially explains the high background in some AMV reverse transcriptases based RT-PCR, especially for quantifying those microRNAs. Interestingly, they also found that the recombinant M-MLVs contain small RNAs derived from *E. coli*. These findings suggest that small RNAs may be co-purified with the enzymes during their production, which is likely to interfere with RNA quantitation by using it as a template or RNA primer for reverse transcription or other unknown mechanisms. The co-purified RNA might come from the non-specific binding of small RNA without appropriate treatment with RNase or the RNA binding activity of reverse transcriptase. —*Qiwei Zha*i	

MicroRNAs (miRNAs) are small RNA molecules (approximately 22 nucleotides long) that take part in the post-transcriptional regulation of gene expression (Chen and Rajewsky, 2007). Thousands of these RNAs have been reported since their discovery. These small molecules have been shown to play an important regulatory role in a wide range of biological and pathological processes (Brennecke et al., 2003; Cuellar and McManus, 2005; Lim et al., 2005). Recently, miRNAs have been found in serum, plasma, urine and other bodily fluids (Chen et al., 2008; Weber et al., 2010). Interestingly, the expression profiles of these extracellular miRNAs are correlated with various diseases; they may be used as biomarkers in diagnosing and monitoring human diseases (Lu et al., 2005).

Various techniques have emerged to quantify miRNAs. MiRNA expression can be quantified by two-step RT-qPCR, which can provide relative quantification (Chen et al., 2005). MiRNAs can also be hybridized on microarrays, slides or chips with probes for hundreds of miRNA targets, and relative miRNA levels can be determined in different samples (Shingara et al., 2005). High-throughput sequencing methods can also be used to profile miRNAs (Buermans et al., 2010). RT-qPCR is usually performed as a standard for accuracy and sensitivity during the typical procedure to determine the miRNA expression profile. For this reason, the quality of the RT-qPCR system is closely related to the final determination and result.

We were surprised to find that the background of certain miRNAs was very high in the RT-qPCR results, even in no-template-controls during practice trials. By considering the high specificity of the stem-loop RT primers, we studied the origin of this high background in miRNA RT-qPCR and found that commercial avian myeloblastosis virus (AMV) reverse transcriptase may contain miRNAs.

In practice, DEPC-treated water or ultrapure water (Invitrogen) served as no-template-controls for miRNA quantitation. However, certain miRNAs (for example, Let-7a, miR-16, miR-26a, and miR-191 in this study) exhibited high background signals in these no-template-controls when AMV reverse transcriptase was used (Fig. 1A). DNA sequencing showed that the PCR products were correct (data not shown). We tried two other commercial AMV reverse transcriptases (purchased from company 2, Co. 2 and company 3, Co. 3) and M-MLV reverse transcriptase (purchased from Co. 1). These miRNAs could also be detected in all AMV reverse transcriptase, but not in M-MLV reverse transcriptase (Fig. 1A). The above results indicated that the miRNA templates were inserted during reverse transcription when using the AMV reverse transcriptase system. Therefore, we tested all the reagents used during RT-qPCR. Our experiments excluded the possibility of miRNA contamination in all the reagents except the AMV reverse transcriptase (data not shown). In fact, only the reverse transcriptase is derived from biologically active substances, which is likely being contaminated during production. We extracted the total RNA from 10 μL or 160 μL of AMV reverse transcriptase (Co. 1), and the total RNA samples served as templates for the reverse transcription reaction; M-MLV reverse transcriptase was used because of its low background. Interestingly, the results showed a dose-dependent pattern in the RT-qPCR reaction (Fig. 1B). The C_T_ values of these miRNAs were found in almost the same Log_2_ proportions as the volumes of reverse transcriptase. These results implied that the miRNAs found in AMV reverse transcriptase might explain the high background signals.

**Figure 1:**
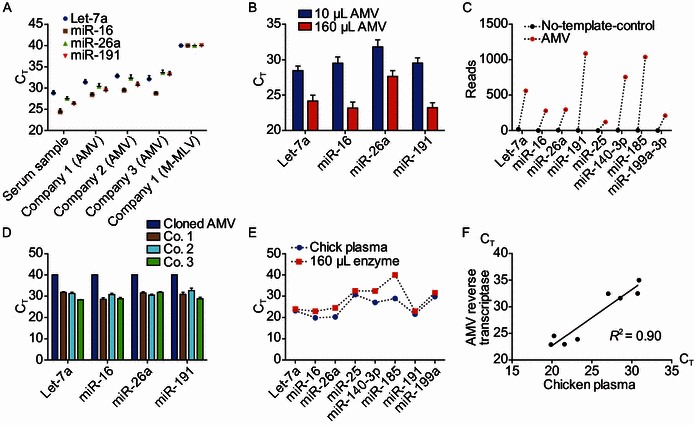
**MiRNAs exists in purified AMV reverse transcriptase**. (A) Four miRNAs reverse transcribed with AMV but not M-MLV from different companies exhibited high background in no-template-control. (B) AMV with higher concentration contained more miRNAs. (C) Solexa sequencing revealed large copy numbers of miRNAs in AMV. (D) MiRNAs expression was found variously in AMV from different companies. (E) and (F) The miRNA profile for chick plasma was consistent with that of the reverse transcriptase (*R*^2^ = 0.90 and *P* value = 0.0003)

Solexa is a next generation sequencing technique, and it was employed in the present study to detect whether the commercial AMV reverse transcriptase solution contained miRNA molecules. Different to the RT-qPCR technique, the RNA samples used for this method were ligated with 5′-adaptors and 3′-adaptors before the reverse transcription reaction. The ligase was then inactivated and reverse transcriptase was added. Afterwards, the approximately 90-bp-long resulting fragments were amplified by PCR (17 cycles) and sequenced. Importantly, only the sequences containing the adaptor sequence were counted as the correct sequences. This procedure guaranteed that the small RNAs within the reverse transcriptase could not be counted in the RNA samples during the ensuing sequence determinations. As shown in the supplementary information, after comparing the sequencing results with an miRNA database, Solexa sequencing revealed hundreds of copy numbers (readings) of many miRNAs were existed in commercial AMV transcriptase (Fig. 1C and Supplemental File 1). The total miRNAs copy number of 8052, miR-191 had a highest copy numbers (1088) and miR-25 had 120 copy numbers, among the eight miRNAs picked up in this assay. To validate these miRNAs by RT-qPCR, the M-MLV RT-qPCR system was employed. Total RNA samples were extracted from 10 μL of commercial AMV reverse transcriptase from three different companies, and a cloned AMV reverse transcriptase (cat. No. 12328-019, Invitrogen) was also extracted as a negative control. As shown in Fig. 1D, four miRNAs were detected in different commercial AMV reverse transcriptases, and the miRNA expression profiles from different companies were diverse. We also detected other miRNAs and found that different AMV reverse transcriptases have slightly different miRNA contents.

To explore the origin of these miRNAs in AMV reverse transcriptase, we traced their source back to the process of AMV reverse transcriptase production. Currently, there are two primary methods used in reverse transcriptase production; one method is to purify AMV reverse transcriptase from avian myeloblastosis virus (AMV) particles isolating from the host plasma or cultured cells infected with AMV (Houts et al., 1979); another method is to purify the enzyme following recombinant expression in *E. coli* or other cells (for example, insect cells). Most commercial AMV reverse transcriptases are produced using the first method. The AMV is used to infect chicks or other birds, after 7 days, AMV particles are purified from the host blood plasma. We checked the miRNA profile of chick plasma by RT-qPCR (Fig. 1E). The miRNA profile for chick plasma was consistent with that of the reverse transcriptase. There were high concentrations of Let-7a, miR-16, miR-26a and miR-191 in chick plasma, and AMV reverse transcriptase also exhibited high expression levels of these miRNAs (Fig. 1E and 1F). Our results suggest that the contaminated miRNA in AMV reverse transcriptase might be derived from host plasma. We suspect that the chick miRNAs are not excluded during AMV reverse transcriptase purification because these miRNAs are binding to the AMV reverse transcriptase.

Commercial M-MLV reverse transcriptase was produced by recombinant protein expression in *E. coli*. As a prokaryotic organism, *E. coli* has no miRNAs. Our results also showed that there is no miRNA in commercial M-MLV reverse transcriptase. However, we wondered whether small RNAs from *E. coli* would contaminate the M-MLV reverse transcriptase, so we extracted the RNA from commercial M-MLV (Co. 1) and performed Solexa sequencing. We found a large quantity of small RNAs (under 50 nucleotides) in the M-MLV reverse transcriptase. The most abundant small RNAs in M-MLV reverse transcriptase (copy numbers > 10000) are listed in Table 1 and Supplemental File 2. BLAST sequencing showed that most of the RNAs were fragments of rRNAs (16S and 23S), and we noted that many small RNA fragments have multiple loci in the *E. coli* genome. The above results indicated that commercial M-MLV reverse transcriptase also contains small RNAs derived from their artificial *E. coli* host. Although these small RNAs may not interfere with miRNA quantification, they could interfere with *E. coli* small RNA identification and characterization.

**Table 1:** Solexa sequencing reveals that MMLV reverse transcriptase contains small RNAs

	Start^a^	End	Ori	Sequence	Copy numbers
1	3418968	3418990	−	ACACCTGATCGTCGAGCTTTACT	29979
2	4014636	4014659	+	AAATTGAAGAGTTTGATCATGGCT	17327
3	223772	223795	+		
4	4145764	4145787	+		
5	4187252	4187275	+		
6	3920913	3920936	+		
7	2709043	2709066	−		
8	3406645	3406668	−		
9	2733502	2733527	+	GGGGCTGATTCTGGATTCGACGGGAT	16212
10	3924793	3924816	+	CCAGGCTGTCTCCACCCGAGACTC	11038
11	4149730	4149753	+		
12	227744	227767	+		
13	4191132	4191155	+		
14	4018609	4018632	+		
15	3402682	3402705	−		
16	2705086	2705109	−		
17	436011	436032	+	TTCCATGATCGCCGGCCTTTTC	10256

^a^The location of small RNAs in *E. coli* genome

We tried to deplete miRNA contamination from AMV reverse transcriptase. We tried to separate the AMV reverse transcriptase and miRNA by filtration. Forty microliters of commercial AMV reverse transcriptase solution was diluted to 500 μL with 1× reaction buffer and added to an Amicon Ultrafiltration tube (>10 kDa, Millipore). After centrifuge, the remaining solutions above the filters were collected. To examine the function of filtration-treated AMV reverse transcriptase, undiluted or diluted transcriptase in different concentrations (100 and 10000 dilution) were used in RT reaction with synthetic miR-16 molecules served as RNA template. To evaluate the miRNAs remaining in the AMV solution, the rest of the solutions were extracted with Trizol to obtain the RNA contents. The extracted RNAs were quantified by RT-qPCR. As shown in Fig. 2A, filtration could decrease the miRNA contamination in AMV solutions: all the C_T_ values of four miRNAs were decreased about 1–2 cycles, which indicates that the miRNAs in AMV solution might be separated from the reverse transcriptase in this condition. Consistently, the function test showed that filtration treatment did not impair the capability of the AMV reverse transcriptase to perform their function in different concentration (Fig. 2B).

**Figure 2:**
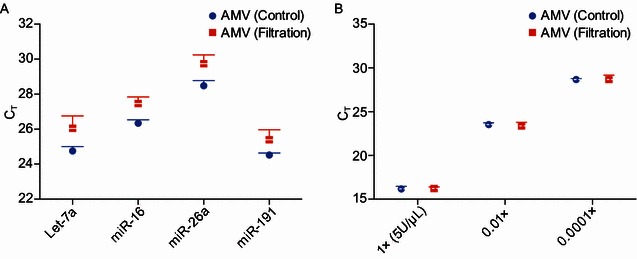
**Filtration decreases the miRNA contents without impairing the capability of the AMV reverse transcriptase**. (A) Filtration could decrease the miRNA contamination in AMV solutions. (B) Filtration treatment did not impair the capability of the AMV reverse transcriptase to perform their function in different concentrations

From the above study, we think that a no-template control is necessary for miRNA quantification using RT-qPCR assay, because of the uncertain miRNA contents of AMV from different sources. With no-template control, an accurate base line of C_T_ value for each miRNA can be figured out. If C_T_ value in experimental group is far below the base line C_T_ value, miRNA contamination from reverse transcriptase may not affect the results; otherwise the possibility of false negative data should be taken into consideration because the high background might conceal the changes of miRNA levels. Although no-template control has not solved the problem of miRNA contamination in reverse transcription, it does provide a reference to get rid of bad data. A reverse transcription procedure is needed when engaging in miRNA sequencing or RT-PCR quantitation. In Solexa, a pair of Solexa adaptors was added to the 5′ and 3′ ends of RNAs to form a 90-bp fragment (small RNA + adaptors) with the help of ligase. The ligase was then inactivated, and reverse transcriptase was added. Afterwards, the 90-bp fragment was amplified through adaptor primers. This procedure guaranteed that the small RNAs present in reverse transcriptase could not be detected during the ensuing sequence determination. We suggest that the strategy of adding adaptors first might be applied to the RT-PCR technique to avoid interference from unwanted miRNAs in future. Choosing M-MLV reverse transcriptase or cloned AMV reverse transcriptase in miRNA studies could eliminate interference from miRNA contamination in reverse transcriptases.

RNase is usually strictly limited during reverse transcriptase production to avoid RNase contamination; otherwise the contaminated RNase will degrade the RNA template in following reverse transcription. Therefore, small RNAs might be co-purified during reverse transcriptase production. According to our results, both AMV and M-MLV reverse transcriptases contain small RNA fragments coming from their host, respectively. During previous studies, we were surprised to find adding synthetic miRNA molecule into reverse transcription system could slightly change the C_T_ value (data not shown), which provided a possibility that small RNA in reverse transcriptase might be a functional co-factor rather than contamination. Although our preliminary studies revealed that partially depleting part of miRNAs in AMV by mild physical methods did not interrupt the function of AMV, we could not exclude the possibility that miRNAs in AMV or small RNAs in M-MLV is a potential co-factor for reverse transcriptase, because filtration could not eliminate all small RNAs in reverse transcriptase. We propose that the small RNAs existing in reverse transcriptase might be functional. Based on our discovery and hypothesis, further studies should focus on the analysis of crystal structure of reverse transcriptase and explore the possible binding site for small RNA.

In summary, small RNAs exist in commercial reverse transcriptase. According to the product description, some commercial AMV reverse transcriptases were derived from avian myeloblastosis virus, which suggested that miRNAs might be derived from the host of the virus particles. Our miRNA profiling study compared the miRNA profile of AMV with the miRNA profile in bird serum. Commercial M-MLV reverse transcriptase and cloned AMV reverse transcriptase are usually produced by *E. coli* strains or in insect cells, which excluded the possibility of mammalian miRNA contamination. Interestingly, Solexa sequencing detected a large number of small RNAs (approximately 20–25 nucleotides) that matched the *E. coli* genome. A special purification step aimed at small RNAs removal might not be included in the production process. All of these problems contribute to high background signal in small RNAs quantification. The uncertain miRNA contamination status of reverse transcriptase would affect data analysis, and it would even prevent certain changes from being detected (false negative). Therefore, the small RNAs in contaminated transcriptase might interfere with quantification in certain cases. This study first identified the interference caused during miRNA RT-qPCR and then identified the source of these miRNAs, providing an optimization protocol for the detection of miRNAs to avoid unnecessary interference during data analysis.

## Electronic supplementary material

Below is the link to the electronic supplementary material.

Supplementary material 1 (DOC 33 kb)

Supplementary material 2 (JPEG 195 kb)

Supplementary material 3 (XLS 40 kb)

Supplementary material 4 (XLS 4616 kb)
