# Supplementary material for: Small RNA existed in commercial reverse transcriptase: primary evidence of functional small RNAs
Source: Protein Cell. 2014 Nov 22;6(1):1–5. doi: 10.1007/s13238-014-0116-2 (PMC4286138; doi:10.1007/s13238-014-0116-2)
Supplement: Supplementary file 1 — Supplementary material 1 (DOC 33 kb) [file 13238_2014_116_MOESM1_ESM.doc]

**MATERIALS AND METHODS**

**Reagents**

DEPC water or ultrapure water, Trizol and miRNA probes were purchased from Life Technologies. dNTP, AMV reverse transcription buffer, and AMV reverse transcriptase were purchased from three different companies (Companies 1-3). Taq polymerase and buffer were purchased from Life Technologies. All other reagents were of analytical grade.

**Human serum samples and experimental chicks**

All human blood samples were collected from healthy patients at the Jinling Hospital (Nanjing, China) at the time of physical examination. All of the donors or their guardians provided written consent, and this study was approved by the Hospital Ethical Examination Committee of Human Research of Nanjing Jinling Hospital, (Nanjing, China) and written informed consent was obtained from each participant. Whole blood was separated into serum and cellular fractions within 2 h after the blood was collected.

Plasma samples were taken from 3 chicks brought from Qianyuan Hao Bio-pharmaceutical companies (Nanjing, China). Seven-day-old chicks were killed, and their plasma was collected. The birds were sacrificed under the NIH Guidelines for the Care and Use of Laboratory Animals, and the animal experiment protocol was approved by the Animal Care Committee of Nanjing University (Nanjing, China).

**RNA isolation and RT-qPCR**

One hundred microliters of human serum was diluted with 300 μl of DEPC water, and the total RNA was extracted with Trizol reagent (Life Technologies, Carlsbad, CA, USA) according to the manufacturer’s instructions. To extract the RNA from commercial reverse transcriptase, 10 μl or 160 μl of AMV solution was diluted with three volumes of DEPC water, and the total RNA was extracted with Trizol reagent. Chick blood was drawn by heart puncture (3~5 ml per chick) into 10 ml negative pressure blood collection tubes containing EDTA potassium salt. Blood cells were sedimented by centrifugation at 3000 g for 10 min, and the upper plasma level was collected. Total RNA was isolated with Trizol reagent (Invitrogen) according to the manufacturer’s instructions. Glycogen (Invitrogen) was added to the RNA precipitation step. The total RNA samples were dissolved in ultrapure water (Life Technologies, Carlsbad, CA, USA). To perform the reverse transcription reaction, 1 μg of total RNA from serum samples or the same volume of ultrapure water (no-template-control) was added to the 10 μl reverse transcription reaction systems (using commercial company products company 1-3). In some experiments, 3 μl of total RNA was extracted and reverse transcriptase was added to the 10 μl reverse transcription reaction systems. A reverse transcription system (TaqMan MicroRNA Reverse Transcription Kit, Applied Biosystems, USA) was used in this study, and it employs a recombinant Moloney murine leukemia virus (rMoMuLV) reverse transcriptase called “MultiScribe Reverse Transcriptase”. This rMoMuLV system can provide a standard reverse transcriptase that is free of miRNA.

Real-time PCR was performed with a TaqMan PCR kit and an Applied Biosystem 7500 Sequence Detection System (Applied Biosystem). All reactions were performed in triplicate, and no-template controls were included. After the reaction, the C_T_ values were determined using fixed threshold settings.

**Solexa sequencing**

The Solexa sequencing was performed by BGI Tech company (China, Shenzhen). Briefly, after the PAGE purification of small RNA molecules and the ligation of a pair of Solexa adaptors to the 5’ and 3’ ends, a 90 base pair fragment (small RNA + adaptor) was amplified by using the adaptor primers for 17 cycles and then isolated on PAGE gels. The isolated DNA sequence was then used for the cluster generation and sequencing analysis using an Illumina Genome Analyzer according to the manufacturer’s instructions. Digital data resulted from the image files generated by the sequencer; a series of procedures were subsequently performed including summarizing data production, evaluating sequencing quality and depth, calculating the length distribution of small RNAs, and filtering out contaminated reads. The clean reads were aligned against the miRBase 16.0 database based on the Smith-Waterman algorithm after constructing the adaptor sequences. Matching miRNAs were counted only after identifying the sequence and length in comparison to reference miRNAs.

**Ultrafiltration**

Amicon Ultra-0.5 Centrifugal Filter Devices (Millipore, 10K) were used to filter the AMV solution. Briefly, 40 μl of AMV solution was diluted to 500 μl with 1 × reaction buffer and added into the Amicon Ultra-0.5 filtration tube (10 kDa). After centrifugation at 14,000 g for 15 minutes, approximately 40 μl of the remaining solution above the filter membrane was collected immediately according to the manufacturer’s instructions. To test the reverse transcription function of filtered AMV, a small aliquot of the samples was used in the RT-qPCR system as the reverse transcriptase (synthetic miR-16 RNA molecules served as the RNA template). The remaining solutions were extracted with Trizol to extract the RNA contents from the AMV. The extracted RNAs were quantified by MMLV-based RT-qPCR. An equal volume of unfiltered AMV was used as a control AMV to perform the miR-16 reverse transcription or RNA extraction and quantification.

**Statistical analysis**

All the RT-qPCR data points are representative of at least three independent experiments and are presented as the means ± SEM. Prism 5.0 software (GraphPad, Inc.) was used for the data analysis.
