# Supplementary figures and images for: Small RNA existed in commercial reverse transcriptase: primary evidence of functional small RNAs
Source: Protein Cell. 2014 Nov 22;6(1):1–5. doi: 10.1007/s13238-014-0116-2 (PMC4286138; doi:10.1007/s13238-014-0116-2)

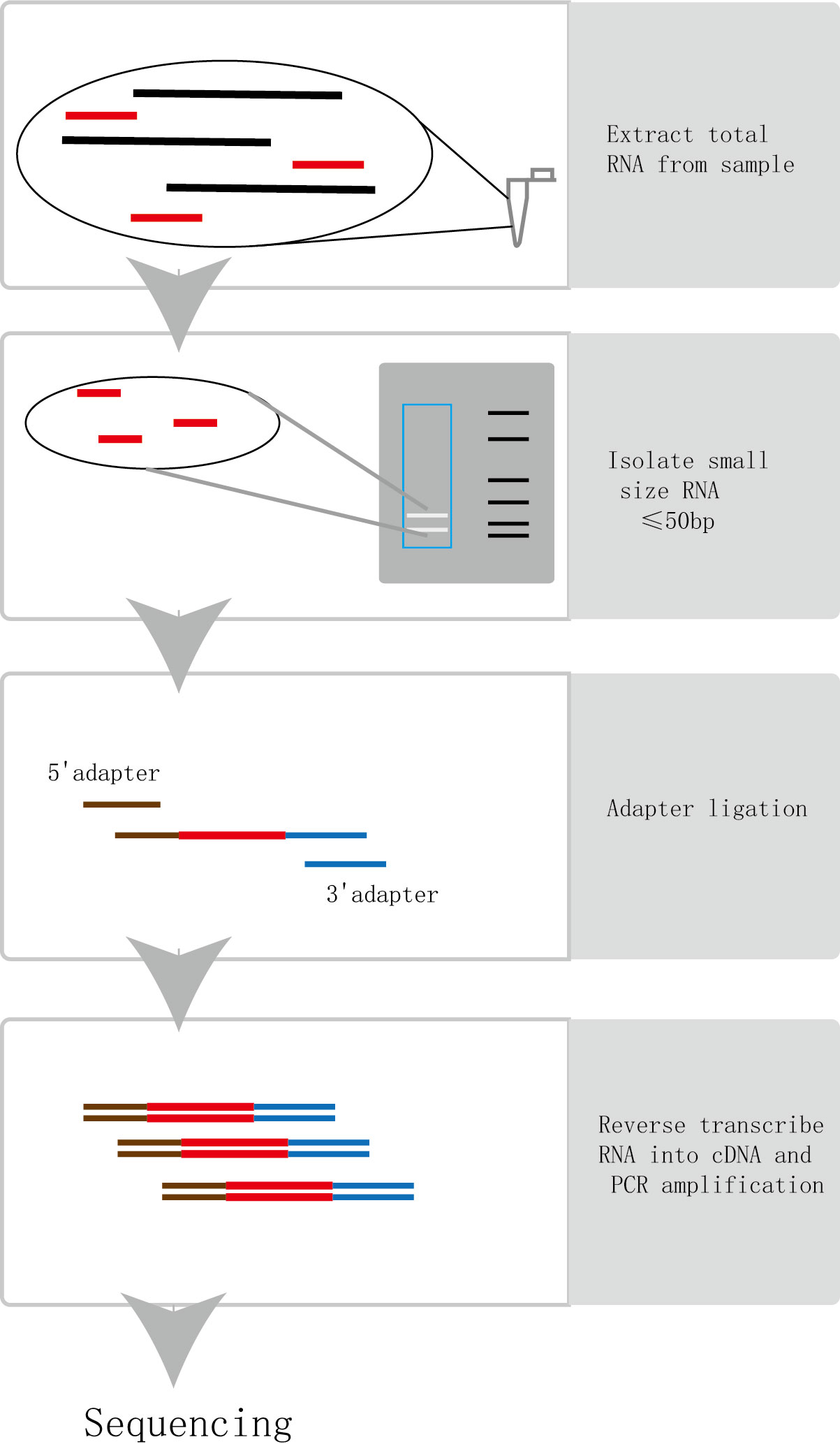

Supplement: Supplementary file 2 — Supplementary material 2 (JPEG 195 kb) [file 13238_2014_116_MOESM2_ESM.jpg]
